# A Rare Presentation of Sarcoidosis in Which a Lady Presented With Massive Splenomegaly and Skin Manifestation Without Lung Involvement: A Case Report

**DOI:** 10.7759/cureus.21020

**Published:** 2022-01-07

**Authors:** Karunakaran Samuel Rajeen, Maheswaran Umakanth

**Affiliations:** 1 Internal Medicine, Teaching Hospital Batticaloa, Batticaloa, LKA; 2 Clinical Medicine, Teaching Hospital Batticaloa, Batticaloa, LKA

**Keywords:** pulmonary interstitium, abdominal pain, isolated, massive splenomegaly, sarcoidosis

## Abstract

Sarcoidosis is a granulomatous disease with multiple system involvement. It is characterized by the presence of non-caseating granulomas in the involved organs. The most commonly affected organ in sarcoidosis is the pulmonary interstitium. However, extra-pulmonary involvement can be manifested in almost any other organ system. Less commonly, sarcoidosis can manifest with massive splenomegaly. As extensive differential diagnoses, such as hematological malignancies, primary splenic or metastatic tumors, infiltrative disorders, and inflammatory disorders are considered, diagnosing sarcoidosis with the presentation of massive splenomegaly is clinically challenging. Here, we discuss the case of a 56-year-old female with splenic sarcoidosis and skin manifestation.

## Introduction

Sarcoidosis is a granulomatous disease with multiple system involvement that is characterized by the presence of non-caseating granulomas in the involved organs [1]. Although the pulmonary interstitium is the most commonly affected organ in sarcoidosis (98%), extra-pulmonary involvements of the skin, eyes, and abdominal organs are not uncommon [1,2]. The second common organ involved is the skin [1]. Isolated extrathoracic sarcoidosis is reported in only 10% of cases [3]. At the same time, approximately 40% of cases of multisystem sarcoidosis have splenic involvement [2]. Sarcoidosis presenting with isolated splenic involvement without any radiographic or clinical pulmonary involvement is very rare. Here, we describe a case of sarcoidosis presenting with isolated splenomegaly in a 56-year-old female patient who presented with constitutional symptoms.

## Case presentation

A 56-year-old woman with a past medical history of diabetes mellitus was admitted after a one-month duration of abdominal distension and two weeks of fever. Her fever was low-grade in nature and was associated with right hypochondrial pain. She had no history of exertional dyspnea, chronic cough, joint pain, or altered bowel habits. Her history did not reveal any risk factors for human immunodeficiency virus, occupational exposure, or exposure to animals.

**Figure 1:**
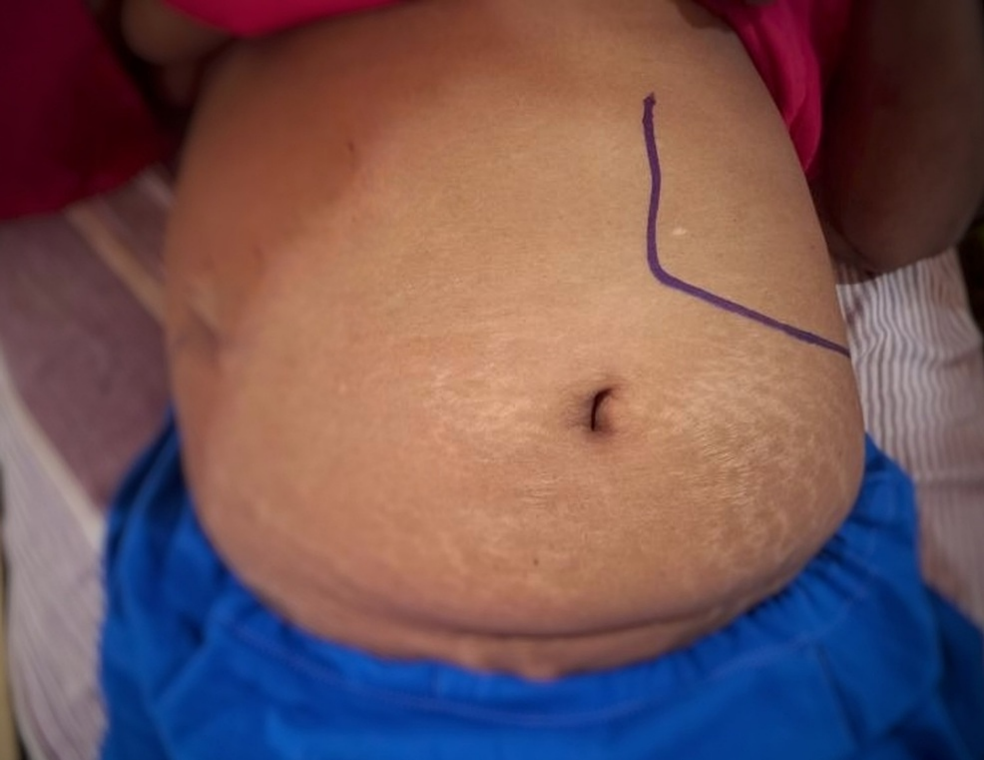
Abdomen examination revealed an enlarged spleen, no hepatomegaly, and no other intra-abdominal masses. Consent was obtained from the patient to take the photograph and publish this image.

The patient was afebrile. Vital signs, including blood pressure, pulse rate, respiratory rate, and oxygen saturation, were within normal ranges. Her systemic examination showed no abnormalities other than in the abdominal system. Upon abdominal examination, an enlarged spleen was detected (Figure 1), but no hepatomegaly or any other intra-abdominal masses were found.

**Figure 2:**
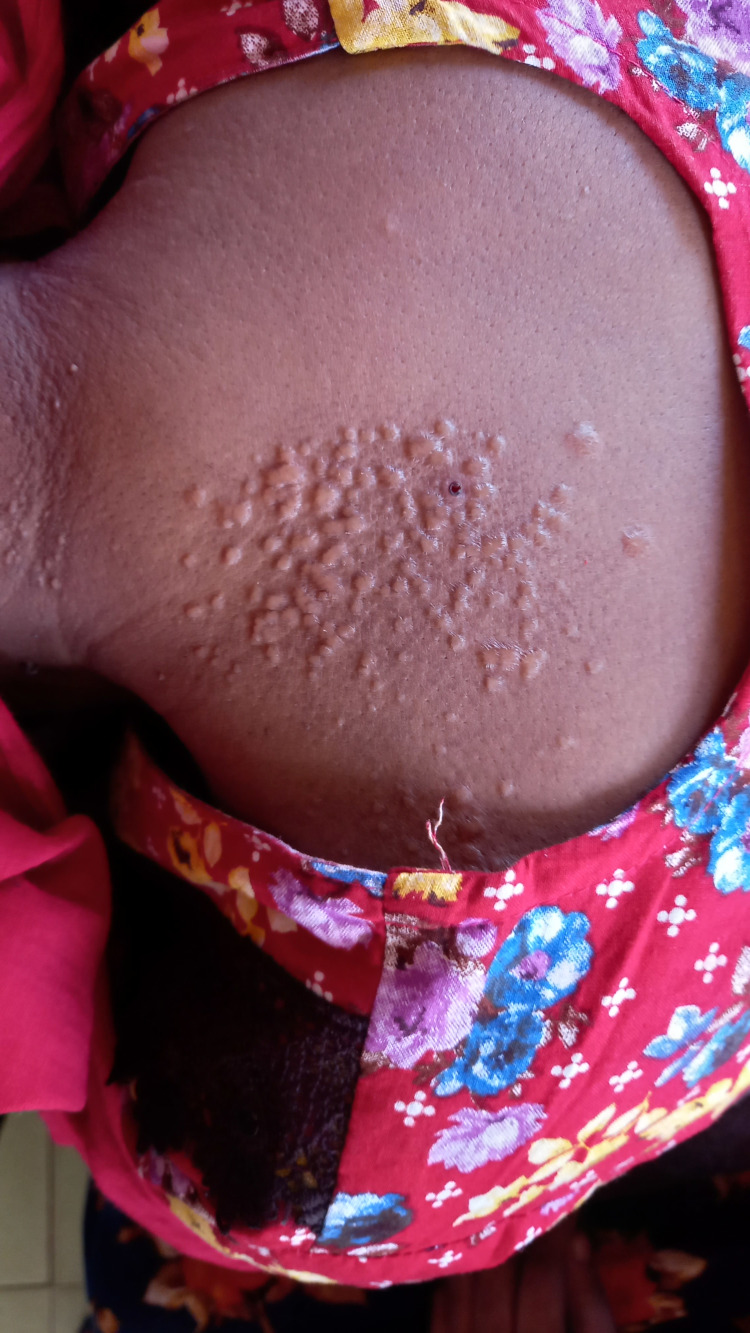
Papular skin rashes over her upper trunk. To publish this image, consent from the patient is obtained.

In addition, the patient had papular skin rashes on her upper trunk (Figure 2).

Laboratory tests showed a white cell count of 14.9 × 103/µl (neutrophils: 8.7 × 103/µl, lymphocytes: 3.69 × 103/µl, eosinophils: 1.37 × 103/µl), hemoglobin of 12.4 g/dl, and platelets of 435 × 103/µl. Her renal function test showed serum creatinine of 69 µmol/l, blood urea of 4.1 mmol/l, serum sodium of 137 mmol/l, and serum potassium of 4.9 mmol/l. SGOT (serum glutamic-oxaloacetic transaminase) was 14 U/L, SGPT (serum glutamic pyruvic transaminase) was 22 U/L, and corrected calcium was 2.4 mmol/L. Her angiotensin-converting enzyme (ACE) level was 228 U/L (Table 1). Urine analysis was normal. A pulmonary function test (PFT) showed normal vital capacity and normal total lung capacity.

**Table 1:** Laboratory test values. WBC: white blood cells, Hb: hemoglobulin, PLT: platelets, SGOT: serum glutamic-oxaloacetic transaminase, SGPT: serum glutamic pyruvic transaminase, ACE: angiotensin-converting enzyme.

	Day 1	Normal range
WBC (10^9^/L)	14.9	4.0–10.0
Neutrophils (10^3^/µl)	8.7	2.0–7.5
Lymphocytes (10^3^/µl)	3.69	1.3–4.0
Eosinophils (10^3^/µl)	1.37	0.0–0.5
Hb (g/dL)	12.4	11.0–15.0
PLT (10^9^/L)	435	150–450
Blood urea (mmol/L)	4.1	1.8–6.3
Serum creatinine (µmol/L)	69	53–88
Serum sodium (mmol/L)	137	136–145
Serum potassium (mmol/L)	4.9	3.5–5.1
SGOT (U/L)	14	15–37
SGPT (U/L)	22	12–78
Corrected calcium (mmol/L)	2.4	2.2–2.6
ACE (U/L)	228	16–85

**Figure 3:**
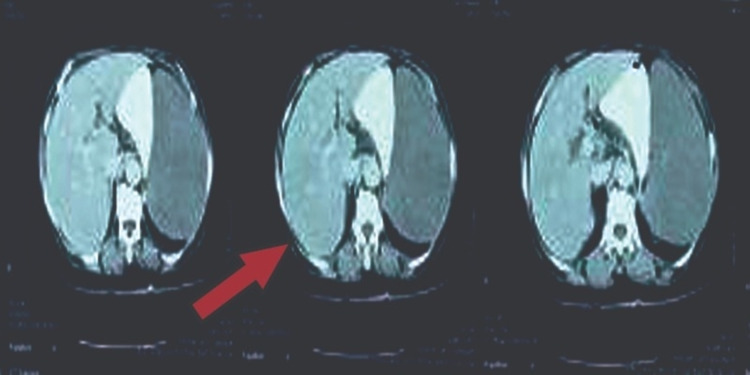
CECT abdomen-pelvis-chest showed massive splenomegaly and no hepatomegaly or liver lesions.

Contrast-enhanced computer tomography (CECT) of the chest, abdomen, and pelvis showed massive splenomegaly (15 cm in size) with no hepatomegaly, liver lesions, or hilar lymph nodes (Figure 3).

**Figure 4:**
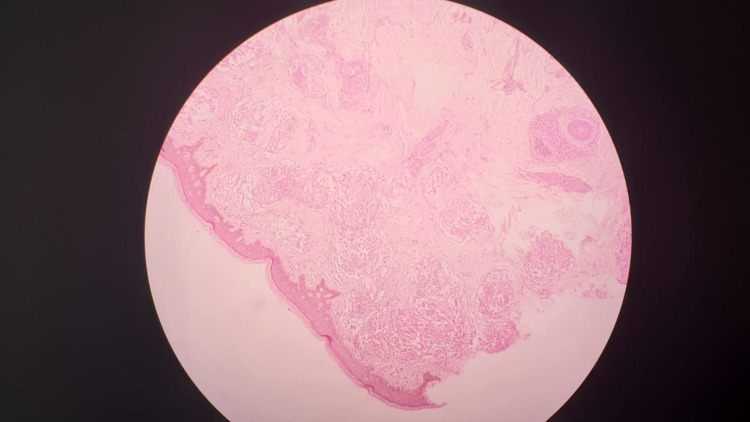
Skin biopsy shows multiple non-caseating closely packed granulomas.

Skin biopsy showed multiple non-caseating closely packed granulomas (Figure 4).

Granulomas are composed of multinucleated giant cells with asteroid bodies. Granulomatous dermatitis favors sarcoidosis. A multidisciplinary team (MDT) approach was employed that included consultant physicians, a respiratory physician, and the dermatologist.

Her electrocardiogram (ECG) did not show any conduction disturbances or arrhythmias, and her 2D echocardiogram (ECHO) revealed no cardiac manifestations of sarcoidosis. Therefore, MDT decided not to go for 18F-fluorodeoxyglucose (FDG) positron emission tomography (PET) and magnetic resonance imaging (MRI). There were no ophthalmological manifestations that indicated sarcoidosis.

Pursuant to the conclusion of the multidisciplinary team, medical management of sarcoidosis was not initiated. Furthermore, the patient was to be followed up at the clinic level to look for any complications, progression of splenomegaly, and any other systemic involvement before deciding on steroid therapy at the clinic level.

## Discussion

Sarcoidosis is a disease of unknown etiology. Clinical manifestations depend on the involved organs. The lungs are the primary organ involved in sarcoidosis, although, rarely, the heart, renal, and gastrointestinal systems can also be involved [4]. Isolated splenomegaly is difficult to diagnose in the absence of clinical suspicion of sarcoidosis. Here, we discuss a case in which sarcoidosis presented with splenomegaly and systemic symptoms of loss of appetite, abdominal pain, and low-grade fever. According to her history, clinical examination, and basic level investigations, we came to the conclusion that the heart, lungs, and kidneys were not involved in this patient.

Usually, the basic laboratory investigations are normal in sarcoidosis [5]. Accordingly, our patient had normal laboratory test results and inflammatory markers. Her ACE level was high. Splenic size and serum ACE levels had a close and linear relationship [6]. However, a normal range of ACE levels has also been reported in different cases.

Radiological imaging plays a vital role in diagnosing atypical presentations of sarcoidosis. In particular, positron emission tomography (PET-CT) and MRI are critical to the assessment of focal lesions in extrapulmonary sarcoidosis. CECT of the chest, abdomen, and pelvis of our patient showed splenomegaly without lung focal lesions and perihilar lymph nodes. Therefore, we excluded lung involvement with the evidence of negative lung function test. Cardiac involvement was excluded by ECG and 2D ECHO, and cardiac MRI and FDG PET scans have recently been used to diagnose cardiac sarcoidosis with better sensitivity and specificity [7]. Retinal involvement was excluded by retinopathy screening.

Since clinical findings and PFT are not suggestive of pulmonary involvement and the CECT chest, abdomen, and pelvis did not show any lung involvement, MDT did not suggest further proceeding with invasive procedures to exclude lung involvement, such as lung biopsy. 

The symptoms and/or findings that necessitate corticosteroid therapy remain controversial. In our patient, medical management of sarcoidosis was not initiated because the patient was asymptomatic and did not show any progression of symptoms or manifestations of complications during the ward stay [8]. However, patients with extrapulmonary sarcoidosis should be evaluated for the progression of systemic symptoms and complications.

## Conclusions

Although sarcoidosis of the spleen is rare, when a patient presents with systemic symptoms and splenomegaly, a differential diagnosis of sarcoidosis should be considered. A patient with sarcoidosis and spleen involvement should be evaluated for lung and other organ involvement during follow-up. Patients with sarcoidosis who have splenic involvement and skin rashes only require immune suppressive treatment with or without topical steroid therapy for skin rashes when they are symptomatic and their symptoms are not resolving spontaneously.
